# Complete genome sequence of *Novosphingobium pentaromativorans* US6-1^T^

**DOI:** 10.1186/s40793-015-0102-1

**Published:** 2015-11-19

**Authors:** Dong Hee Choi, Yong Min Kwon, Kae Kyoung Kwon, Sang-Jin Kim

**Affiliations:** Marine Biotechnology Research Division, Korea Institute of Ocean Science and Technology, Ansan, 426-744 Republic of Korea; Major of Marine Biotechnology, University of Science and Technology, Daejeon, 305-350 Republic of Korea; National Marine Biodiversity Institute of Korea, Seocheon, 325-902 Republic of Korea

**Keywords:** Polycyclic aromatic hydrocarbon, *Novosphingobium*, Megaplasmids, Extradiol dioxygenase

## Abstract

*Novosphingobium pentaromativorans* US6-1^T^ is a species in the family *Sphingomonadaceae*. According to the phylogenetic analysis based on 16S rRNA gene sequence of the *N. pentaromativorans* US6-1^T^ and nine genome-sequenced strains in the genus *Novosphingobium*, the similarity ranged from 93.9 to 99.9 % and the highest similarity was found with *Novosphingobium* sp. PP1Y (99.9 %), whereas the ANI value based on genomes ranged from 70.9 to 93 % and the highest value was 93 %. This microorganism was isolated from muddy coastal bay sediments where the environment is heavily polluted by polycyclic aromatic hydrocarbons (PAHs). It was previously shown to be capable of degrading multiple PAHs, including benzo[a]pyrene. To further understand the PAH biodegradation pathways the previous draft genome of this microorganism was revised to obtain a complete genome using Illumina MiSeq and PacBio platform. The genome of strain US6-1^T^ consists of 5,457,578 bp, which includes the 3,979,506 bp chromosome and five megaplasmids. It comprises 5110 protein-coding genes and 82 RNA genes. Here, we provide an analysis of the complete genome sequence which enables the identification of new characteristics of this strain.

## Introduction

The polycyclic aromatic hydrocarbons are widely distributed in the environment as one of the persistent organic pollutants and are generated by natural combustion processes as well as human activities [1]. Benzo(a)pyrene is of environmental concern due to its high carcinogenic [2] and bioaccumulation potential [3]. Biodegradation in contaminated environments is one of the important processes of remediation. Therefore, isolation of potent biodegradation strains and elucidation of the biodegradation pathways have drawn attention for a long time [4–6]. *Novosphingobium pentaromativorans* US6-1^T^, a Gram negative halophilic marine bacterium, is one of the potent strains capable of utilizing a series of high molecular weight PAHs as sole carbon and energy sources. Strain US6-1^T^ showed an especially high degradation ability for benzo(a)pyrene [7]. To understand the PAH biodegradation pathways, genomic and proteomic approaches were conducted on this strain [8, 9]. In the genomic study it was reported that strain US6-1^T^ contained at least two large plasmids and most of the coding genes associated with PAH degradation were located in the larger plasmid pLA1 [8]. However, the draft genome sequence was inadequate to understanding the degradation processes for high-molecular-weight compounds of PAH and their regulation mechanism. Therefore, completion of the strain US6-1^T^ genome was carried-out and the genomic repertoire is reported in here.

## Organism information

### Classification and features

**Fig. 1:**
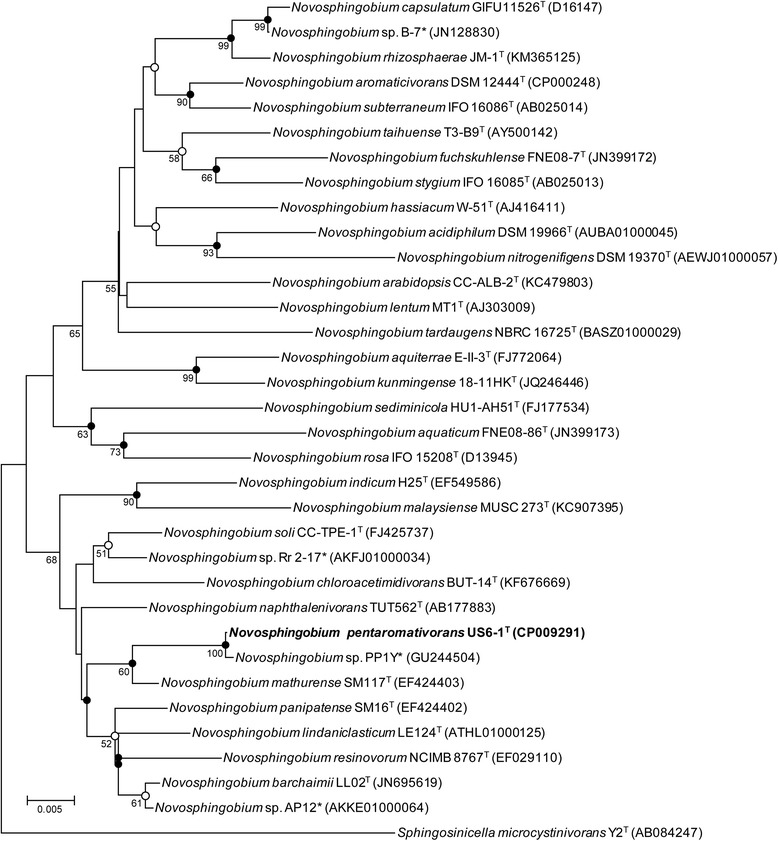
Phylogenetic tree highlighting the position of *Novosphingobium pentaromativorans* US6-1^T^ (in bold) relative to the other validly published 28 type strains, and 4 non-type strains that have their whole genome sequences (indicated with *) within genus *Novosphingobium*. A total of 1305 unambiguously aligned sequences were compared and phylogenetic trees were reconstructed using the neighbor-joining [26], maximum-likelihood [27] and maximum-parsimony [28] methods. Bootstrap values (%) are based on 1000 replicates and are indicated at the nodes when they are higher than 50 % [29]. The evolutionary distances were calculated by the Jukes-Cantor method [30] using MEGA5 [31]. The nodes are marked with filled or open circles when the node was recovered by all three or by two treeing methods, respectively. *Sphingosinicella microcystinivorans* Y2^T^ was used as an outgroup. Scale bar; 0.005 changes per nucleotide position

At the time of writing, the genus *Novosphingobium* contains 30 species including *N. pentaromativorans* US6-1^T^. Phylogenetic analysis based on the 16S rRNA gene sequences using the neighbor-joining, maximum-likelihood and maximum-parsimony methods showed that *N. pentaromativorans* US6-1^T^ formed a clade with other members within the genus *Novosphingobium* (Fig. 1). *N. pentaromativorans* US6-1^T^ shared the 16S rRNA gene identity with the type strains, *N. aquaticum* FNE08-86^T^ and *N. mathurense* SM117^T^, in the range of 93.9 and 98.7 %, respectively. The strain PP1Y [10], one of the whole-genome sequenced strains in genus *Novosphingobium*, was most closely related to *N. pentaromativorans* US6-1^T^ with 99.9 % similarity.

Strain US6-1^T^ cells are Gram-negative, non-motile rods (Table 1). Cells are 0.36–0.45 μm in width and 0.97–1.95 μm in length. Colonies on ZoBell 2216 agar and trypticase soy agar medium are yellowish and circular. Optimal growth occurred at 30 °C and was retarded below 20 °C. The organism tolerates pH values from 6 to 9 and optimal growth occurs at pH 6.5. Strain US6-1^T^ grows in the range of 1–6 % NaCl with optimal growth at 2.5 % NaCl. The isolate can grow under anaerobic conditions but growth is retarded [7].

**Table 1:** Classification and general features of *N. pentaromativorans* US6-1^T^

MIGS ID	Property	Term	Evidence code^a^
	Current classification	Domain *Bacteria*	TAS [33]
		Phylum *Proteobacteria*	TAS [34]
		Class *Alphaproteobacteria*	TAS [35, 36]
		Order *Sphingomonadales*	TAS [36, 37]
		Family *Sphingomonadaceae*	TAS [38, 39]
		Genus *Novosphingobium*	TAS [40, 41]
		Species *Novosphingobium pentaromativorans*	TAS [7]
		Type strain US6-1^T^	TAS [7]
	Gram stain	negative	TAS [7]
	Cell shape	rod	TAS [7]
	Motility	non-motile	TAS [7]
	Sporulation	not reported	NAS
	Temperature range	15-40 °C	IDA [7]
	Optimum temperature	30 °C	TAS [7]
	pH range; Optimum	6–9; 6.5	TAS [7]
	Carbon source	cyclodextrin, dextrin, glucose, maltose, sucrose, psicose, propionic acid,alanine, glutamic acid, proline	TAS [7]
MIGS-6	Habitat	muddy sediment	TAS [7]
MIGS-6.3	Salinity	requires (2.5 %)	TAS [7]
MIGS-22	Oxygen requirement	Facultative anaerobic	TAS [7]
MIGS-15	Biotic relationship	free-living	TAS [7]
MIGS-14	Pathogenicity	non-pathogen	TAS [7]
MIGS-4	Geographic location	Ulsan Bay, Republic of Korea	TAS [7]
MIGS-5	Sample collection time	2000	NAS
MIGS-4.1	Latitude	129°23′14″	NAS
MIGS-4.2	Longitude	35°29′48.5″N	NAS
MIGS-4.4	Altitude	−8 m	NAS

^a^Evidence codes - IDA: Inferred from Direct Assay; TAS: Traceable Author Statement (i.e., a direct report exists in the literature); NAS: Non-traceable Author Statement (i.e., not directly observed for the living, isolated sample, but based on a generally accepted property for the species, or anecdotal evidence). These evidence codes are from the Gene Ontology project [42]

*N. pentaromativorans* US6-1^T^ utilizes cyclodextrin, dextrin, Tween 40, Tween 80, α-D-glucose, maltose, D-trehalose, sucrose, psicose, methyl pyruvate, β-hydroxybutyric acid, α-ketobutyric acid, propionic acid, acetic acid, quinic acid, L-alanine, L-alanyl glycine, L-aspartic acid, L-glutamic acid, L-proline, L-threonine and L-phenylalanine [7]. These phenotypes were confirmed by genomic methods.

## Genome sequencing information

### Genome project history

The genome of *N. pentaromativorans* US6-1^T^ was sequenced in 2009 using a 454 GS FLX Titanium sequencing platform. The assembly and annotation of draft genome sequences were completed on August 11, 2011 and the GenBank data was released on September 5, 2011. The genome project has been deposited at DDBJ/EMBL/GenBank under the accession number AGFM00000000 [8]. On January 1, 2014, *N. pentaromativorans* US6-1^T^ was selected for complete genome sequencing using Illumina MiSeq and PacBio RS II sequencing technology. The complete genome was annotated on May 26, 2014 by ChunLab Inc., South Korea and the sequence was deposited in GenBank on October 10, 2014 (CP009291, CP009292, CP009293, CP009294, CP009295, CP009296). Table 2 represents the project information and its association with MIGS version 2.0 compliance [11].

**Table 2:** Project information

MIGS ID	Property	Term
MIGS-31	Finishing quality	Finished
MIGS-28	Libraries used	Illumina MiSeq, PacBio 10 K
MIGS-29	Sequencing platforms	Illumina MiSeq, PacBio 10 K
MIGS-31.2	Fold coverage	395.08 × Illumina, 128.82 × PacBio
MIGS-30	Assemblers	Roche gsAssembler 2.6, PacBio SMRT
		Analysis 2.2.0, CLCbio CLC Genomics
		Workbench version 7.0.4
MIGS-32	Gene calling method	Prodigal, tRNA-Scan-SE, HMMER
	Locus Tag	JI59
	GenBank ID	CP009291-6
	GenBank Date of Release	October 10, 2014
	GOLD ID	Gs0114422
	BIOPROJECT	PRJNA257352
MIG-13	Source Material Identifier	KCTC 10454^T^
	Project relevance	Bioremediation, PAHs biodegradation pathway, Environmental

### Growth conditions and genomic DNA preparation

US6-1^T^ (=KCTC 10454^T^) was cultivated for 1 day at 30 °C in 100 ml ZoBell medium (5 g peptone, 1 g yeast extract, 0.01 g FePO_4_ per liter of 20 % distilled water and 80 % filtered aged seawater) by shaking incubation (150 rpm). Cell was harvested by centrifugation at 6000 × g for 15 min at 4 °C and then washed twice with sterilized seawater. The genomic DNA isolation prepared by using a Wizard® genomic DNA purification kit (Promega, USA) according to the manufacturer’s instructions. Genomic DNA quantified using the PicoGreen® fluometric quantification kit (Molecular Probes) and preserved at −20 °C for sequencing.

### Genome sequencing and assembly

The genomic DNA was fragmented using dsDNA fragmentase to generate DNA pieces suitable for library construction. The DNA fragments were processed with a TruSeq DNA sample preparation kit v2 (Illumina Inc., USA) following the manufacturer’s instructions. The final library was quantified by a Bioanalyzer 2100 (Agilent, USA) and the average library size was 300 bp. The genomic library was sequenced by Illumina MiSeq (Illumina Inc., USA) and a PacBio RS II sequencer (Pacific Biosciences, USA). Generated Illumina sequencing reads (8,767,104 reads, total read length 2,156,191,562 bp) and PacBio reads (1,362,072 reads, total read length 703,045,197 bp) were assembled using the CLC genomics workbench 7.0.4 (CLC bio, Denmark) and the PacBio SMRT Analysis Pipeline 2.2.0. Finally, we obtained 6 contigs. The contigs and PCR-based long reads were combined through manual curation using CodonCode Aligner 3.7.1 (CodonCode Corp., USA). The final plasmid sequences were corrected by remapping with raw reads to check errors and dubious regions.

### Genome annotation

The genes in the assembled genome were predicted using Prodigal [12] as part of the DOE-JGI genome annotation pipeline [13, 14], followed by a round of manual curation using the JGI GenePRIMP pipeline [15]. tRNAs were identified by tRNA-Scan-SE [16], and the search for rRNAs used HMMER with EzTaxon-e rRNA profiles [17, 18]. The predicted CDSs were compared to catalytic families, NCBI COG by rpsBLAST, NCBI reference sequences and SEED databases by BLASTP, for functional annotation [19–22]. Additional gene prediction analysis and functional annotation were performed within the Integrated Microbial Genomes-Expert Review (IMG-ER) platform [23].

## Genome properties

**Fig. 2:**
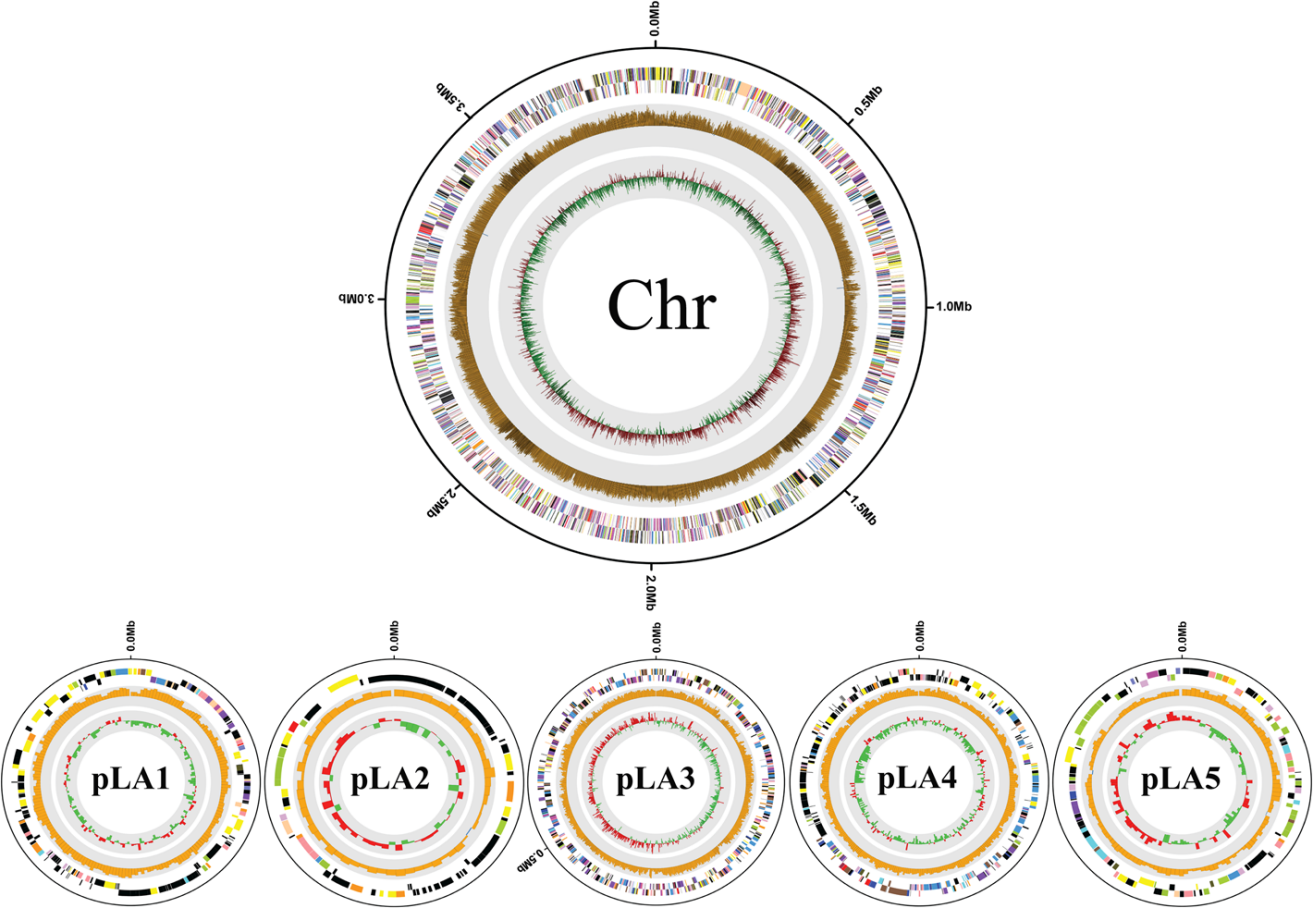
Circular maps and genetic features of the chromosome and its plasmids of *N. pentaromativorans* US6-1^T^ displaying relevant genome features. From outside to center; Genes on forward strand (colored by COG categories), genes on reverse strand (colored by COG categories), GC content and GC skew. Order and size counterclockwise from an upper map: Chr, 3.98 Mb; pLA 1, 0.18 Mb; pLA 2, 0.06 Mb; pLA 3, 0.75 Mb; pLA 4, 0.33 Mb; pLA 5, 0.13 Mb

The total length of the complete genome sequence is 5,457,578 bp, which includes a 3,979,506 bp chromosome and five plasmids pLA 1 (0.18 Mb), pLA 2 (0.06 Mb), pLA 3 (0.75 Mb), pLA 4 (0.33 Mb), and pLA 5 (0.13 Mb) (Table 3). The DNA G + C content was determined to be 63.02 %. There are 82 RNA genes which includes 9 rRNAs, 54 tRNAs and 19 miscRNAs (Table 4). All of the amino acid coding genes are located on the chromosome. From the gene prediction results, 5110 CDSs were identified. The statistics of the genome based on the IMG (ID: 59347) are summarized in Table 4 and the distribution of genes into COG functional categories is presented in Fig. 2 and Table 5.

**Table 3:** Summary of genome: one chromosome and five plasmids

Label	Size (Mb)	GC (%)	No. genes	Topology	INSDC identifier
Chromosome	3.98	63.5	3811	circular	CP009291
pLA1	0.18	62.6	191	circular	CP009294
pLA2	0.06	60.29	85	circular	CP009296
pLA3	0.75	61.44	654	circular	CP009292
pLA4	0.33	62.4	326	circular	CP009293
pLA5	0.13	61.06	125	circular	CP009295

**Table 4:** Genome statistics

Attribute	Value	% of total^a^
Genome size (bp)	5,457,578	100.00
DNA coding (bp)	4,910,346	89.97
DNA G + C (bp)	3,439,297	63.02
DNA scaffolds	6	100.00
Total genes	5192	100.00
Protein coding genes	5110	98.42
RNA genes	82	1.58
Pseudo genes	59	1.14
Genes in internal clusters	4183	80.57
Genes with function prediction	4036	77.73
Genes assigned to COGs	3787	72.94
Genes with Pfam domains	4124	79.43
Genes with signal peptides	486	9.36
Genes with transmembrane helices	1073	20.67
CRISPR repeats	0	0

^a^The total is based on either the size of the genome in base pairs or the total number of protein coding genes in the annotated genome

**Table 5:** Number of genes associated with general COG functional categories

Code	Value	% age	Description
J	167	3.1	Translation, ribosomal structure and biogenesis
A	1	0.0	RNA processing and modification
K	267	4.9	Transcription
L	289	5.3	Replication, recombination and repair
B	0	0.0	Chromatin structure and dynamics
D	36	0.7	Cell cycle control, Cell division, chromosome partitioning
V	50	0.9	Defense mechanisms
T	122	2.2	Signal transduction mechanisms
M	245	4.5	Cell wall/membrane/envelope biogenesis
N	64	1.2	Cell motility
U	76	1.4	Intracellular trafficking and secretion
O	172	3.1	Posttranslational modification, protein turnover, chaperones
C	294	5.4	Energy production and conversion
G	177	3.2	Carbohydrate transport and metabolism
E	272	5.4	Amino acid transport and metabolism
F	67	3.2	Nucleotide transport and metabolism
H	131	5.0	Coenzyme transport and metabolism
I	260	4.8	Lipid transport and metabolism
P	264	4.8	Inorganic ion transport and metabolism
Q	100	1.8	Secondary metabolite biosynthesis, transport and catabolism
R	382	7.0	General function prediction only
S	351	6.4	Function unknown
-	1676	30.7	Not in COGs

The total is based on the total number of protein coding genes in the annotated genome

## Insights from the genome sequence

**Fig. 3:**
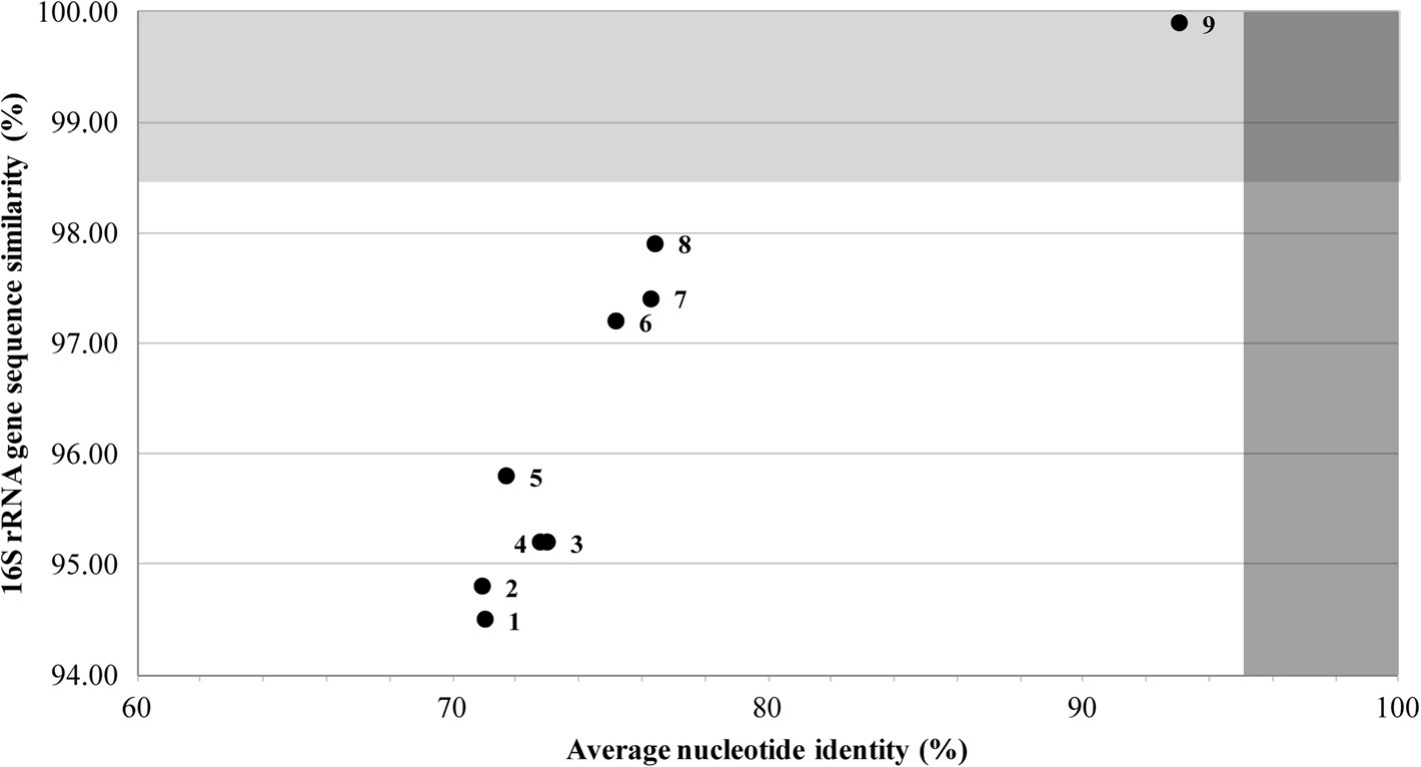
The relationship between 16S rRNA gene sequence similarities and ANI values for strains in the genus of *Novosphingobium*. The species boundary of 16S rRNA gene sequence similarity and ANI value are indicated at 97–98.65 % [24] and 95–96 % [32], respectively. 1, *N. acidiphilum* DSM 19966^T^; 2, *N. tardaugens* NBRC 16725^T^; 3, *N. aromaticivorans* DSM 12444^T^; 4, *Novosphingobium* sp. B-7; 5, *N. nitrogenifigens* DSM 19370^T^; 6, *Novosphingobium* sp. Rr 2-17; 7, *N. lindaniclasticum* LE124^T^; 8, *Novosphingobium* sp. AP12; 9, *Novosphingobium* sp. PP1Y

In this study, the relationship between 16S rRNA gene sequence similarity and ANI value of the *N. pentaromativorans* US6-1^T^ was examined for nine genome-sequenced strains in the genus *Novosphingobium*. The 16S rRNA gene sequence similarity ranged from 93.9 to 99.9 % whereas the ANI values ranged from 70.9 to 93 % (Fig. 3). All interspecies relations (plot number 1–8 in Fig. 3) coincided with the species delineation, while the relation (plot number 9 in Fig. 3) between *N. pentaromativorans* US6-1^T^ and *Novosphingobium* sp. PP1Y showed the discrepancy of the species delineation in terms of 16S rRNA gene sequence similarities and ANI values. This evidence suggests that the strains US6-1^T^ and PP1Y are likely different species, because ANI (93 %) is lower than 95 % in spite of the 99.9 % 16S rRNA gene sequence similarity [24]. However, Gan et al. [25] demonstrated that these two strains may belong to the same species on the basis of average amino acid identity, dinucleotide relative abundance values and genome signature dissimilarity. Kim et al. [24] reported several exceptional cases of the proposed standard for species delineation. Among them a high number of cases (39 %) with >98.65 % 16S rRNA gene sequence similarity, and <95 % ANI, were found for strains that are known to have high intraspecific or intragenomic variations between multiple 16S rRNA genes in the genome. The same case was found between *N. pentaromativorans* US6-1^T^ and *Novosphingobium* sp. PP1Y in the current study even though the intraspecific or intragenomic variations between multiple 16S rRNA genes in those genomes were low. At present, it is not clear how 16S rRNA gene sequence similarity between these two strains has been conserved despite having relatively divergent genomes.

Strain US6-1^T^ has two different extradiol pathways [9]. A previous analysis found that genes involved in the catechol 2,3-dioxygenase pathway are encoded in plasmid pLA1, whereas those of the protocatechuate 4,5-dioxygenase pathway are located in the chromosomal genome. Based on the completed genome data, however, it was discovered that most of the protocatechuate 4,5-dioxygenase genes are encoded in pLA3 (three alpha-subunits and two beta-subunits are in pLA3, with one beta-subunit in the chromosome) and that both extradiol biodegradation pathways are encoded separately in two plasmids. Additional gene such as a copy of naphthalene 1,2-dioxygenase involved in aromatic hydrocarbon degradation is encoded in the chromosomal genome.

## Conclusions

*N. pentaromativorans* US6-1^T^ was isolated from marine sediments and it showed halophilic characteristics. This strain is capable of degrading multi-ring aromatic compounds including benzo[a]pyrene. By completing the genome sequencing, the genomic composition of *N. pentaromativorans* US6-1^T^ was revised from one chromosome and two plasmids to one chromosome and five plasmids, and the total size was changed from approximately 5.1 to 5.5 Mb. The relationship between 16S rRNA gene sequence similarities and ANI values of the *N. pentaromativorans* US6-1^T^ and nine genome-sequenced strains in the genus *Novosphingobium* indicated that all interspecies relations coincided with the species delineation, while the relation between *N. pentaromativorans* US6-1^T^ and *Novosphingobium* sp. PP1Y did not. The two extradiol pathways are distributed on two of the plasmids and some dioxygenase genes such as a copy of protocatechuate 4,5-dioxygenase beta-subunit and naphthalene 1,2-dioxygenase genes involved in aromatic hydrocarbon degradation are encoded in chromosomal DNA. The current findings using this complete genome sequence of *N. pentaromativorans* US6-1^T^ show that the PAHs biodegradation pathway genes are distributed on two plasmids. This result differs from the findings of the draft genome sequence we previously reported [8]. Further research is required to reveal the full pathway of high-molecular-mass aromatic hydrocarbon degradation and its regulation mechanism.

## Abbreviations

ANI: Average nucleotide identity
PAHs: Polycyclic aromatic hydrocarbons
